# Influence of Ukraine war on the foreign medical students

**DOI:** 10.5339/qmj.2024.66

**Published:** 2024-11-11

**Authors:** Suha Turkmen, Salma Kahal, Kamal Majed, Ahmed Ahmed, Isma Qureshi, Zohaer Khan, Kamran Khan, Maha Al-kurbi, Serdar Karakulukcu

**Affiliations:** 1 Department of Emergency Medicine, Qatar University, Doha, Qatar; 2 Department of Emergency Medicine, Hamad Medical Corporation, Doha, Qatar *Email: drsuhaturkmen@hotmail.com; 3 Kharkiv National Medical University, Kharkiv, Ukraine; 4 Hamad Medical Corporation, Department of Emergency Medicine, Doha, Qatar; 5 Hamad Medical Corporation, Department of Emergency Medicine, Doha, Qatar; 6 Hamad Medical Corporation, Department of Emergency Medicine, Doha, Qatar; 7 Qatar University Collage of Medicine, Doha, Qatar; 8 Karadeniz Technical University, School of Medicine, Department of Public Health, Trabzon, Turkiye

**Keywords:** Medical education, foreign students, Ukraine, war

## Abstract

**Background:**

Wars are escalating globally with devastating impacts on all aspects of life. The conflict between Ukraine and Russia began on February 24, 2022. Approximately 80,000 students of 155 nationalities were studying in Ukraine when the war started, disrupting their education and forcing many to leave the country. We aimed to determine the physical, emotional, and moral effects of the Ukrainian war on foreign medical students, as well as the secondary impacts of the war on the students due to the ongoing conflict.

**Methods:**

The participants were non-Ukrainian medical students aged 18 years and over, studying at a medical school in Ukraine before the war started. A survey including the depression anxiety stress scales-21 (DASS-21) scale variables, a validated and reliable measure of depression, anxiety, and stress dimensions, and other questions on participants’ demographics, education, and current socio-economic status was sent to all eligible students via their registered university emails and distributed using an online link.

**Results:**

A total of 99 students were included in the study. 52 (52.5%) of the students were female and 49 (49.5%) were between the ages of 23 and 24 years old. Participants reported high levels of depression (86.9%) and anxiety (82.8%), with significant percentages experiencing extreme levels: 40.4% for depression and 55.6% for anxiety. Additionally, 74.7% reported feeling stressed, with 18.2% indicating extreme stress. Reasons for leaving Ukraine included safety concerns (67.7%), seeking a more secure educational environment (63.6%), the impact of the ongoing war and conflict on their future (56.6%), and the loss of educational opportunities (28.3%).

**Conclusion:**

It is not easy to predict how the war in Ukraine will affect the education of international students in the near future. This uncertainty situation may explain students’ depression, anxiety, and stress. As a result, it is necessary to design effective strategies to maintain the training of health professionals during wartime. Research should be conducted on how to rebuild health education systems after the wartime crises stabilize, both for students who are citizens of the country exposed to war and for foreign students who went to that country to receive education, and solutions for this should be put forward.

## 1. Background

Wars in the world are increasing dramatically, and before one end, a new war breaks out in another part of the world. It is obvious that wars have devastating effects on all aspects of life. From a human health perspective, wars have direct effects such as ending lives, multiple casualties, serious injuries, permanent disabilities, and psychological effects. However, there are also indirect effects such as increase in infectious diseases, malnutrition, diminished access to care, medications, violence against medical personnel, damage to health care facilities, and collapse of health care delivery system, which are very difficult to compensate for and perhaps impossible for a long time.^1,2^ Another indirect consequence is its influence on medical education. Training healthcare professionals is essential for fostering a skilled workforce that plays a vital role in sustaining and improving the healthcare system. Wars not only cause delays, reductions, or complete cessation of education and training programs for health professionals, but also result in the departure of qualified health professionals. Considering these complex interactions, the effects of war do extend well beyond the active war period.^3^

When the war started between Ukraine and Russia on February 24, 2022, there were 23 medical schools in Ukraine. These schools did not only have students from Ukraine, but students coming from all over the world to receive medical education. Ukrainian universities, whose medical diplomas are accepted all over the world, including the World Health Organization and the Council of Europe, were a popular location for students who could not receive medical education in their own countries for various reasons.^4^ When the war broke out, approximately 80,000 students of 155 different nationalities were receiving education at different grades and in different universities.^5^ With the start of the war, not only their education was interrupted, but the students were also forced to leave the country. This only compromised their ongoing education, but they were left with uncertainty related to their futures.^5^

At the beginning of the war, the students struggled to survive and took shelter in safe places. Most of them tried to return to their country safely, thinking that it would not be possible to continue their education in the chaos of the war environment. They faced and still have to face many challenges in their efforts to continue their education. Students who had to take a break from their regular classroom education at first tried to continue their education with alternative methods such as online education. However, due to reasons such as bombardment, power outages, and internet outages, online training was disrupted and bedside clinical training, which is indispensable for medical education, could not be carried out.^6^ Medical educators were also affected by the war environment, and the increased workload severely limited the time they could devote to education. As a result of the war, the decrease in financial resources of educational institutions and all these interconnected factors caused the quality of education to decrease. There were many students who tried to continue their medical education in alternative countries and institutions. Some of them were able to find this opportunity, but for most of them there is still uncertainty, and they have serious concerns about whether they will be able to graduate.^7^

Therefore, we aimed to examine how the war in Ukraine directly or indirectly affected foreign medical students physically, emotionally, and morally. We also tried to explore the effects of the war on their education and various aspects of their lives. Furthermore, our study aimed to understand the secondary effects of the ongoing conflict on these students.

## 2. Methods

### 2.1. Setting

This study is a prospective cross-sectional online survey conducted among foreign medical students who were affected by the ongoing conflict in Ukraine. The study was conducted from October 1 to December 1, 2023.

### 2.2. Study design and participants

The participants were non-Ukrainian medical students aged 18 years and over, studying at a medical school in Ukraine before the war started. The questionnaire was sent to as many students as possible from Ukrainian medical schools. The students have been approached via their registered emails that are in records in their universities. The questionnaire was distributed through an online link, and a form was created to collect data from the participants. The form was designed in such a format, where the initial page opened as a research information sheet, explaining the objectives, methods, outcomes, risks, and withdrawal procedure of the study. Once the participant agreed to participate, by clicking on the “willing to participate” button, they were directed to the next page containing details of the questions related to the survey. The participants had the opportunity to skip questions. After the completion of the form, they had to click on the “complete and submit” button and the response was automatically incorporated into an Excel sheet. There was no risk related to this study; however, the participant might have felt uncomfortable completing the survey about their mental health status and recalling any negative experiences related to the Ukrainian war and related challenges. Once the participants submitted their response, their participation could not be withdrawn as the data collection were done anonymously.

Ethical approval was obtained from the Institutional Review Board (IRB) of the Medical Research Center, Hamad Medical Corporation, Qatar, Doha (MRC-01-23-503). The study was conducted along the local and international guidelines for survey-based studies.

### 2.3. Data collection

The questionnaire incorporates depression anxiety stress scales-21 (DASS-21) scale variables. The scale is a valid and reliable measure of the dimensions of depression, anxiety, and stress.^8^ It also includes a more general dimension of psychological stress. It is a self-reporting instrument, and no special skills are required to score it. The DASS is available in two forms, 21 and 42 items, respectively. However, each is rated on a 4-point scale of how much each statement applies to the person. We opted for DASS-21 because we believed it would be more practical for our study. Higher scores will indicate a greater psychological impact due to war. The questionnaire also included questions about the demographics, education, and current socio-economic status of the participants.

### 2.4. Sample size

The study sample was calculated as at least 65 students in the G Power 3.1.9 program with a 95% confidence interval, 90% power, and an effect size of 0.20. It was observed that the sample size was similar in previous studies.^6^ There were no incentives or rewards given to the participants.

### 2.5. Statistical analysis

SPSS 24.0 statistical package program was used during the data analysis phase. Descriptive statistics of evaluation results: for categorical variables, numbers and percentages are given, and for numerical variables, mean, standard deviation, median, and 1st and 3rd quarters (Q1–Q3) are given. The suitability of the groups for normal distribution was determined using the Kolmogorov-Smirnov test. Comparisons of numerical variables between two independent groups were evaluated with the independent samples T-test when the normal distribution condition was met and with the Mann-Whitney U test when it was not met. Comparisons of numerical variables between three independent groups were evaluated with the one-way ANOVA test when the normal distribution condition was met and with the Kruskal-Wallis test when it was not met. If significant differences are found in the Kruskal-Wallis test, pairwise comparisons will be tested using the Bonferroni correction. Statistical alpha significance level was accepted as p < 0.05.

## 3. Results

**Figure 1.**
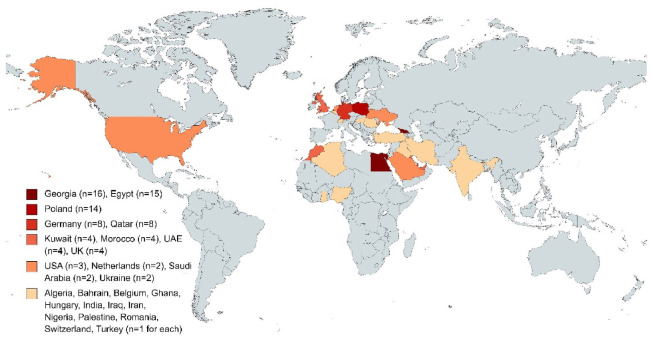
Distribution of countries where medical students currently live after the Ukraine war.

A total of 99 students participated in the study. Among them, 52 (52.5%) were female and 49 (49.5%) were between the ages of 23 and 24 years old. The sociodemographic characteristics of the students are shown in Table 1. When we look at the distribution of the countries where students currently live, the highest number is Georgia, Egypt, and Poland. More than half of the students (n = 59; 59.6%) were studying at Kharkiv National Medical School (Figure 1).

The distributions of student answers to war/conflict and career questions are shown in Table 2. Among the war/conflict questions, it was answered that the city where the war took place was most affected (94.9%), and among the career questions, it was answered that education was most affected (78.8%).

**Figure 2.**
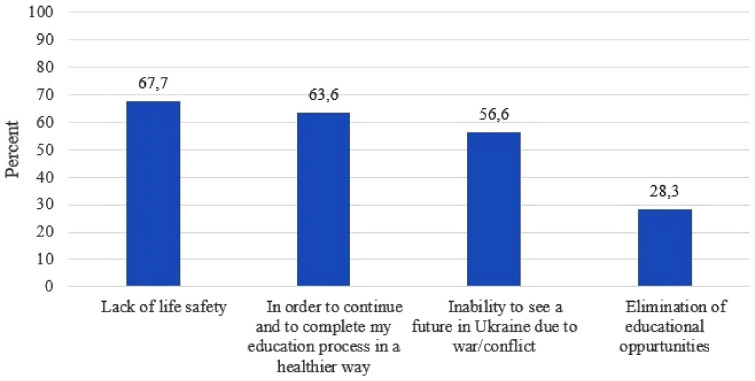
Reasons why medical students left Ukraine after the Ukrainian war.

According to the results of our study, 67 students (67.7%) left Ukraine due to a lack of security, 63 students (63.6%) because they could not continue their education in a stable way, 56 students (56.6%) because of uncertainty about their future in Ukraine due to the ongoing war, and 28 students (28.3%) because of the loss of educational opportunities (Figure 2).

When evaluated according to their DASS-21 scale scores, the students were found to have extreme rates of 40.4% for depression, 55.6% for anxiety, and 18.2% for stress (Table 3). The comparison of DASS-21 scale scores based on students’ sociodemographic characteristics is shown in Table 4. The anxiety scores of students who had an income-generating job during the war (24.8 ± 11.2) were found to be statistically significant higher than those who did not (18.9 ± 10.2) (*p* = 0.034). Depression scores of those who stayed in hotel-pension (median [IQR] 32 [26–34]) were found to be significantly higher than those who stayed at home (median [IQR] 22 [12.5–29.5]) (*p* = 0.038).

The comparison of DASS-21 scale scores based on student responses to questions about war/conflict and career is summarized in Table 5. Depression, anxiety, and stress scores were found to be statistically significantly higher in those whose homes were affected by the war, in those whose friends/relatives participated in the war, in those whose friends/relatives were affected, and in those whose friends/relatives died (*p* < 0.05). In the career questions, the anxiety scores of those who had the opportunity to receive special training or gain experience in working with communities affected by war/conflict were found to be statistically higher (*p* = 0.002), while the stress scores of those whose war/conflict negatively affected their perception of the role of healthcare professionals in Ukraine were higher than the scores of those who were not affected and were found to be high.

## 4. Discussion

Our results showed that a large portion of the students included in our study tried to continue their education in Georgia and Egypt (Figure 1). Georgia was thought to be the most popular place due to its proximity to Ukraine, both geographically and in terms of socio-cultural structure. Additionally, Georgia was seen as an attractive option for students due to the similarities between the two countries in terms of access to medical education. Most medical schools in Georgia are accredited and recognized in all European countries. Egypt has also become a frequent destination for students. Due to the war, the Egyptian government allowed Egyptian citizens to be admitted to medical schools in their country. Therefore, students who traveled to Egypt were thought to take advantage of this opportunity. Similarly, in other studies, medical students and professionals had to migrate to other countries due to security problems and to continue their education due to the war that broke out in their countries while they were studying medicine, and it was observed that a large number of them migrated to European countries.^9^ Students may have chosen European countries because of their proximity to Ukraine and the prospect of receiving high-quality and modern medical education there. According to our results, 67.7% of the students left due to a lack of life safety, and 90% did not consider returning to Ukraine. The negative consequences of the war on medical education are well known and explained. In another study, it was stated that because of the war in their countries, many students fled the war and settled in other countries or had to abandon medical education due to inequalities in the programs and political and social reasons.^10-13^ In our study, it was observed that all foreign students left the country due to the war, and 78.8% of them did not have the opportunity to continue their careers yet. There were many students who wanted to continue their education in European countries. When students came to Europe, they faced many difficulties in obtaining documents and integrating into society. The first thing students noticed was the inability of foreign students to obtain legal status, discriminatory treatment, and then the language barrier. All of this greatly affected the students’ mental health. The most common symptoms among students who knew they were not accepted to medical education were stress and anxiety. Some students contemplated suicide due to the uncertainty about their future. Many students who were accepted into universities for a short period did not want to leave the universities they had reached in Europe and wished to continue their education there. However, many of them did not have the opportunity to continue their education in Europe (Figure 1).

Another impression we got from our study results is that a large portion of the students stated that their education was affected by the war (Table 2). In other studies, it is understood that universities show differences in terms of being affected by war due to their locations. It was observed that students were less affected by the effects of the war in the part that was close to the European border and away from the conflict area. Although their education was affected, another study conducted while the war was ongoing evaluated the educational satisfaction of students, and this study showed that satisfaction did not decrease significantly during the war.^14^ The effective factor here is that distance education experiences and habits during the COVID-19 process before the war helped students to continue their education. During the war, tools and applications such as open-source learning platforms, course management systems, video communication services, data sharing, and social media networks continued to be used.^15^ It has been stated that educational satisfaction may not have changed much compared to the pre-war period.^15^ However, in case of war, there are many different elements from the COVID process. Examples of these include electricity and internet outages during the war. It is obvious that these factors will also affect distance education opportunities.

Another effect of the war that was observed was career planning in regard to the selection of their choice of specialty. We saw that 70% of the students changed their decisions regarding their field of specialization. While we did not specifically inquire about their preferred specialty in our study, other studies have highlighted that medical students’ exposure to and interest in emergency medicine tend to increase during times of conflict.^16^

Wars share three basic characteristics with other disasters. First, it causes injury and death to a significant number of people. Second, it disrupts services, access to resources, and social interactions, and third, it causes psychological and physical health problems in the population.^17,18^ The impact of war on the mental health of the population is well described in the literature, and its consequences can affect active soldiers, women, children, and elderly patients, and in general, every individual can be mentally affected.^19-23^ Moreover, experience from past armed conflicts and wars has shown that this effect can last for decades after the man-made disaster.^24,25^ It was found that 88.9% of the people included in the study were affected by the current conflict.

According to our results, 86.9% of the participants were depressed, 82.8% were anxious, and 74.7% were stressed (Table 3). When armed conflict directly affects the home, the physical integrity of friends, or the life, the psychological effects of war were significantly greater for these three variables. At this point, it should be noted that there may be differences between students who experience the effects of war closely and those who experience it remotely. Another result we observed in our study was that gender did not affect the degree of depression, anxiety, and stress (Table 4). Other studies have shown different results. Depression and anxiety were reported as more common in women.^4,26,27^ In their study on the psychological distress of medical students during the conflicts in Syria, Al Saadi et al. found that female medical students were twice as likely to be depressed and anxious as men.^22^ This difference can be explained by the difference in demographic characteristics between the two study groups. Our study population was not homogeneous, and all our students had different backgrounds and cultures.

In our study, medical students from the 4th to the 6th grades were more likely to suffer from depression, anxiety, and stress, without a statistically significant difference. The lack of a statistically significant difference can be explained by the relatively small sample size in our study. Other studies have shown that depression is more common in the first years of war and in times of peace.^27-29^ Students in the final years of the medical curriculum are assumed to be better able to control stressful factors, but in times of war this ability is less likely to overcome the misconception of being so close to graduation and not completing medical school. Students living at home were less depressed but equally anxious and stressed. Family support and living together may be factors that prevent depression. These results are consistent with other research findings.^29,30^

Students who work in a paying job are more likely to be anxious but not depressed or stressed. The fact that people have incomes and the fear of losing these incomes due to war may explain our study results. There are different results in the literature on this issue. Some previous studies have argued that having money-earning jobs and fear of losing these incomes during war negatively affect people’s psychology, while other studies have argued that this has little effect on people’s psychology in the event of war.^17^

## 5. Limitations

This study specifically targeted a student population, which raises concerns about the generalizability of the findings to other groups, such as faculty or non-student populations affected by the conflict. As participants were asked to reflect on their mental health and wartime experiences, the responses collected may have been affected by recall bias. Furthermore, the retrospective nature of the study may have resulted in participants selectively recalling events or emotions that overlapped with their current feelings, resulting in recall bias. Participants may also have tended to present themselves more positively or to avoid addressing some questions. While these limitations are inherent and recognized in survey-based research conducted in conflict settings, they are unavoidable given the circumstances.^31^

## 6. Conclusions

As a result, the war negatively affected medical education in Ukraine. It is not easy to predict how the war in Ukraine will affect the education of international students in the near future. Most of the students who came for education fell into uncertainty. This state of uncertainty explains students’ depression, anxiety, and stress. The results of our study showed that the war had a negative impact on students’ career plans and took away the opportunity for many of them to continue their medical education. Our results add other important considerations to the literature that highlight the need for a deep discussion to secure the future of healthcare actors in the context of real political and armed conflicts. These findings can also guide education leaders, international aid agencies, and donor organizations to optimize support to effectively sustain health professionals education efforts in times of war. After war crises stabilize, research should be conducted on how to rebuild health education systems for both students who are citizens of the country exposed to war and foreign students who come to that country to receive education, and solutions for this should be put forward.

## Conflicts of Interest Statement

None.

## Authors’ Contributions

ST, SK, AA: Conceptualization; Writing—original draft preparation; Data curation; Formal analysis; Methodology; Project administration. KM, IQ, SK: Formal analysis; Writing—review & editing. ZK, KK, MA-k: Conceptualization; Resources; Supervision; Validation; Writing—review & editing.

## Authorship Declaration

All authors are in agreement with the content of the manuscript.

## Figures and Tables

**Table 1.** Sociodemographic characteristics of students.

**Variables**	***n***	**%**
**Age (year)**		
18–20	3	3.0
21–22	24	24.2
23–24	49	49.5
25–26	23	23.2
**Gender**		
Male	47	47.5
Female	52	52.5
**Marital Status**		
Single	95	96.0
Married	4	4.0
**Current year of study**		
1	2	2.0
2	3	3.0
3	10	10.1
4	16	16.2
5	46	46.5
6	22	22.2
**Income generating job**		
Yes	18	18.2
No	79	79.8
No response	2	2.0
**Total income**		
Less than $1000/per month	55	55.6
More than $1000/per month	18	18.2
No response	26	26.2
**Accommodation**		
Home	92	93.0
Hotel	4	4.0
Pension	3	3.0
Living status		
Alone	30	30.3
With Family	38	38.4
With Friends	31	31.3

**Table 2.** Distribution of students’ answers to war/conflict and career questions.

**War/conflict questions**	**Reply**	***n***	**%**
Have you been affected by the war?	Yes	88	88.9
	Mild	1	1.0
	Moderate	4	4.0
	Severe	20	20.2
	Extreme	63	63.6
Has your city been affected by the war?	Yes	94	94.9
Has your home been affected by the war?	Yes	64	64.6
Did your friends or relatives participate in the war?	Yes	50	50.5
Has your friend or relative been affected by the war?	Yes	36	36.4
Did your friend or relative die in the war?	Yes	26	26.3
**Career questions**			
Has your education been affected by the war?	Yes	78	78.8
Do you think about going back to Ukraine?	Yes	10	10.1
Have you had the opportunity to receive training or experience in working with war/conflict affected communities?	Yes	38	38.3
	No	57	57.5
	No response	4	4
Did the war/conflict change your decision to specialize in a different specialty?	Yes	71	71.7
	No	26	26.2
	No response	2	2
How has the war/conflict affected your perceptions of the role of health workers in Ukraine?	Positive	32	32.3
	Negative	49	49.5
	No Change	18	18.2

**Table 3.** Evaluation of students according to DASS-21 scale scores.^8^

	**Normal *n* (%)**	**Mild *n* (%)**	**Moderate *n* (%)**	**Severe *n* (%)**	**Extreme *n* (%)**
Depression	13 (13.1)	10 (10.1)	20 (20.2)	16 (16.2)	40 (40.4)
Anxiety	17 (17.2)	2 (2.0)	13 (13.1)	12 (12.1)	55 (55.6)
Stress	25 (25.3)	7 (7.1)	18 (18.2)	31 (31.3)	18 (18.2)

**Table 4.** Comparison of DASS21 scale scores according to students’ sociodemographic characteristics.

		**Depression**	**Anxiety**	**Stress**
Gender	Female	22.2 ± 11.5	21 (12–26)	22 (12–28)
	Male	21.5 ± 11.2	27 (16–32)	24 (12–30)
	*p*	0.786	0.576	0.226
Age (year)	18–22	19.3 ± 13.0	18.7 ± 12.1	20 (8–30)
	23–26	22.8 ± 10.5	20.0 ± 10.4	26 (16–31.5)
	*p*	0.174	0.608	0.162
Current year of study	1–3	14 (10–18)	18 (12–22)	16 (10–28)
	4–6	24 (16–32)	22 (12–27.5)	26 (16.5–31.5)
	*p*	0.093	0.304	0.192
Income generating job	Yes	27 (18.5–35)	24.8 ± 11.2	27 (19.5–38)
Response (*n* = 97)	No	22 (14–30)	18.9 ± 10.2	24 (14–30)
No response (*n* = 2)	*p*	0.094	**0.034**	0.128
Total income	Less than $1,000/per month	21.1 ± 11.5	18.5 ± 10.7	24 (12–30)
Response (*n* = 74)	More than $1,000/per month	24.0 ± 12.0	21.9 ± 11.8	26 (13.5–36.5)
No response (*n* = 25)	*p*	0.352	0.267	0.394
Accommodation	Home	22 (12.5–29.5)	21 (12–26)	24 (12.5–30)
	Hotel-pension	32 (26–34)	24 (18–30)	30 (20–36)
	*p*	**0.038**	0.356	0.125
Living status	Alone	23.3 ± 11.5	25 (12–30.5)	22.3 ± 11.8
	With family	21.5 ± 12.0	27 (16–33)	24.0 ± 11.4
	With friends	20.8 ± 10.3	24 (12–30)	22.2 ± 10.8
	*p*	0.675^†^	0.737^¥^	0.760^¥^

Values are given as Mean ± SD or Median (Q1–Q3) according to normal distribution conditions. When normal distribution was achieved, *T* test was used in independent groups, and when not, Mann-Whitney U test was used.

^†^ One-way ANOVA test.

^¥^ Kruskal-Wallis test. Statistically significant comparisons are shown as bold values.

**Table 5.** Comparison of DASS21 scale scores according to students’ answers to war/conflict and career questions.^8^

		**Depression**	**Anxiety**	**Stress**
War/conflict questions				
Have you been affected by the war?	Yes	26 (18–32)	22 (16–27.5)	26 (20–32)
	No	2 (0–2)	2 (0–4)	2 (0–6)
	*p*	**<0.001**	**<0.001**	**<0.001**
Has your city been affected by the war?	Yes	22 (14–30)	22 (12–26)	24 (14–30)
	No	26 (13–34)	24 (10–25)	30 (13–31)
	*p*	0.527	0.981	0.737
Has your home been affected by the war?	Yes	28 (18–32)	22.5 ± 10.2	25.3 ± 10.5
	No	18 (6–26)	14.4 ± 10.0	18.7 ± 11.5
	*p*	**0.001**	**<0.001**	**0.005**
Did your friends or relatives participate in the war?	Yes	25.6 ± 9.2	24 (18–28)	24.8 ± 9.2
	No	20.2 ± 12.5	14 (4–24)	18.9 ± 12.4
	*p*	**0.015**	**<0.001**	**0.009**
Has your friend or relative been affected by the war?	Yes	26 (20–33.5)	24 (18.5–31)	28 (20–36)
	No	20 (12–28)	16 (8–24)	22 (12–30)
	*p*	**0.031**	**0.001**	**0.007**
Did your friend or relative die in the war?	Yes	28 (22–34)	25 (22–33)	28 (23.5–36.5)
	No	20 (11–28)	18 (8–24)	22 (12–30)
	*p*	**0.003**	**<0.001**	**0.003**
Career questions				
Has your education been affected by the war?	Yes	24 (14–32)	19.9 ± 10.7	26 (15.5–30.5)
	No	18 (10–28)	18.6 ± 11.6	24 (10–29)
	*p*	0.061	0.607	0.356
Do you think about going back to Ukraine?	Yes	29 (19.5–34)	22.2 ± 10.8	27 (24–32)
	No	22 (13–30)	19.4 ± 10.8	24 (13–30)
	*p*	0.157	0.436	0.224
Have you had the opportunity to receive training or experience in working with war/conflict affected communities?	Yes	23 (15.5–32)	23.7 ± 9.2	26 (19–31)
Response (*n* = 95)	No	22 (12–31)	16.7 ± 11.3	20 (12–30)
No response (*n* = 4)	*p*	0.510	**0.002**	0.266
Did the war/conflict change your decision to specialize in a different specialty?	Yes	26 (14–32)	20.1 ± 10.8	24 (14–30)
Response (*n* = 97)	No	18 (11.5–29)	18.2 ± 11.2	24 (11.5–28.5)
No response (*n* = 2)	*p*	0.140	0.449	0.493
How has the war/conflict affected your perceptions of the role of health workers in Ukraine?	Positive	19.9 ± 11.2	22 (12.5–26)	26 (12–29.5)^a^
	Negative	24.1 ± 10.5	22 (14–28)	26 (18–33)^a^
	No change	19.2 ± 12.9	13 (3.5–24)	20 (6–28.5)^b^
	*p*	0.149^†^	0.081^¥^	**0.048** ^¥^

Values are given as Mean ± SD or Median (Q1–Q3) according to normal distribution conditions. When normal distribution was achieved, T test was used in independent groups, and when not, Mann-Whitney U test was used.

^a-b^ shows the groups with statistically significant differences.

^†^ One-way ANOVA test.

^¥^ Kruskal-Wallis test. Statistically significant comparisons are shown as bold values.
